# Emergent Biosensing Technologies Based on Fluorescence Spectroscopy and Surface Plasmon Resonance

**DOI:** 10.3390/s21030906

**Published:** 2021-01-29

**Authors:** Alessandra Camarca, Antonio Varriale, Alessandro Capo, Angela Pennacchio, Alessia Calabrese, Cristina Giannattasio, Carlos Murillo Almuzara, Sabato D’Auria, Maria Staiano

**Affiliations:** 1 Institute of Food Science, CNR Italy, 83100 Avellino, Italy; alessandra.camarca@isa.cnr.it (A.C.); antonio.varriale@isa.cnr.it (A.V.); alessandro.capo@isa.cnr.it (A.C.); angela.pennacchio@isa.cnr.it (A.P.); alessia.calabrese@isa.cnr.it (A.C.); cristina.giannattasio@isa.cnr.it (C.G.); carlos.murillo-almuzara@stud.sbg.ac.at (C.M.A.); maria.staiano@isa.cnr.it (M.S.); 2 URT-ISA at Department of Biology, University of Naples Federico II, 80126 Napoli, Italy

**Keywords:** biosensor, SPR, fluorescence, food, security, health, environment

## Abstract

The purpose of this work is to provide an exhaustive overview of the emerging biosensor technologies for the detection of analytes of interest for food, environment, security, and health. Over the years, biosensors have acquired increasing importance in a wide range of applications due to synergistic studies of various scientific disciplines, determining their great commercial potential and revealing how nanotechnology and biotechnology can be strictly connected. In the present scenario, biosensors have increased their detection limit and sensitivity unthinkable until a few years ago. The most widely used biosensors are optical-based devices such as surface plasmon resonance (SPR)-based biosensors and fluorescence-based biosensors. Here, we will review them by highlighting how the progress in their design and development could impact our daily life.

## 1. Introduction

Today the wide selection of available biosensors results to be segmented both on the bases of utilization and technology. Based on the used sensing technology, the extended array of biosensors can be classified into the following groups: (a) electrochemical biosensors; (b) optical biosensors; (c) piezoelectric biosensors; (d) thermal biosensors, and (e) nanomechanical biosensors.

Firstly, it is useful to restate that a biosensor can be considered as an analytical device incorporating a biological sensing element able to specifically bind to a substrate and turn this event into a measurable and quantifiable signal.

**Figure 1:**
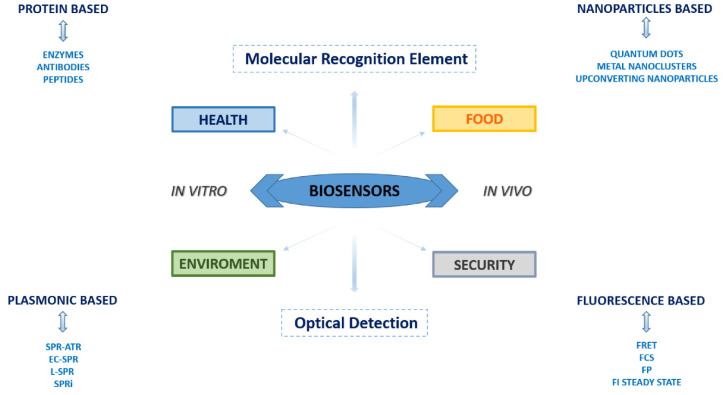
Schematic representation of the disclosed topics.

Usually, a biosensor device results composed of at least of three principal elements: (1) a “biological element” that recognizes the molecular target and, consequently, upon the binding of the target molecule, it generates a detectable signal; (2) a “transducer” that is able to highlight the generated signal; (3) an amplifier, that is able to quantify and transfer the signal to the operator (see Figure 1).

**Figure 2:**
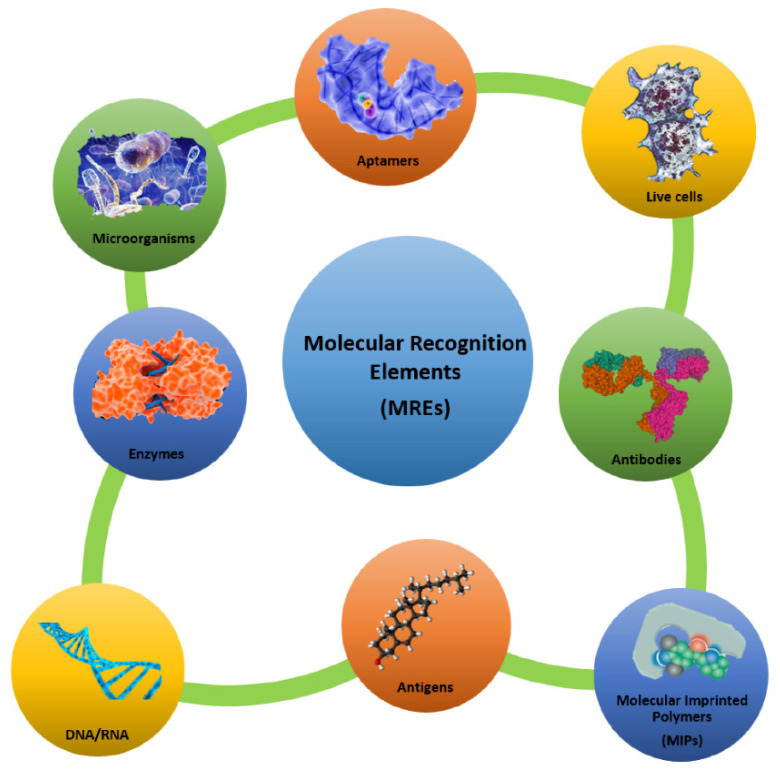
Molecular recognition elements—an overview. A selective overview of molecular recognition elements: protein receptors, enzymes, antibodies, nucleic acids, molecular imprinting polymer, cells, microorganisms, and aptamers.

The use of an appropriate biological sensing element such as an enzyme, a protein, a nucleic acid sequence, an antibody, a microorganism, a part of a tissue, a cell, etc. is the most important step in the design of a biosensor. In fact, biological molecules possess special structural and functional features (such as high specificity and selectivity towards a target substrate), and they provide numerous advantages if used as molecular recognition elements (MREs) (see Figure 2). In addition, it is possible to overexpress them in vector systems to obtain large amounts of recombinant biomolecules, and it is also possible to genetically manipulate them for improving their structural and/or functional properties.

In order to be successful whatever the nature and the quantity of the target analyte to be measured, a biosensor must possess at least some of the following features: (1) the biological sensing element must be highly specific for the target analyte; (2) it should be stable respect to some physical parameters such as pH and temperature variations; (3) it should be able to measure target analytes in complex real matrices with marginal pre-treatment steps of the sample; (4) the sensing response should be fast, accurate and reproducible (especially referred for early detection and diagnostics analyses); (5) it should be easily miniaturized and easy to use by semi-skilled operators [1].

Developing new biosensors that possess the above-described properties is of great relevance since they can be applied for tracing contamination and/or manipulation occurring in the food marketplace, such as foodborne pathogens, toxins of different origin, antioxidants, preservatives, and other potentially dangerous chemicals. Biosensors are a precious tool also for monitoring environmental pollutants and toxic molecules (ranging from pesticides and herbicides, to aromatic compounds, and metal ions) dispersed in the atmosphere, water, and soil. Issues related to human security are also increasing in the last decade due to terrorist threats. Consequently, devices able to detect the presence of explosive substances are required in places like ports and airports, arenas, and institutional or government buildings. Finally, in the health field, biosensors are nowadays ubiquitous, being spread across biomedical research and clinical practice. The rapid and precise detection of many analytes, ranging from molecular disease-associated biomarkers to inflammation mediators, small metabolites, neurotransmitters, hormones, enzymes, etc., have crucial importance in terms of basic disease knowledge, as well as of drug design and diagnostics.

Here, we will review the state-of-the-art of surface plasmon resonance and fluorescence-based biosensors reporting their recent applications in the above-mentioned fields. For the health section, a focus on in vivo applications for research purposes, is also provided.

## 2. Surface Plasmon Resonance (SPR) Based Biosensors

**Figure 3:**
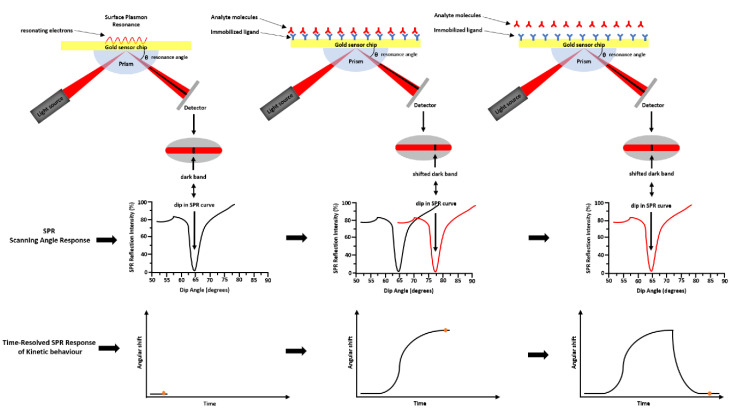
Surface plasmon resonance—ATR via the Kretschmann configuration. Light is focused onto a metal film through a glass prism and the subsequent reflection is detected. At a certain incident angle (or resonance angle), the plasmons resonate at the same light frequency, resulting in the absorption of light at that angle. This determines a dark line in the reflected beam. That dark line contains a wealth of information. The resonance angle can be obtained by observing a dip in SPR reflection intensity. A shift in the reflectivity curve represents a molecular binding event taking place on or near the metal film, or a conformational change in the molecules bound to the film. By monitoring, this shift vs. time is possible to study the molecular binding events and binding kinetics.

SPR is a technique based on the opto-electronic phenomenon that occurs when a visible or near-infrared light is incident upon a metal surface, such as Ag, Au, Cu, and Al. The radiation will through a specific prism and collimated to a detector (photodiode array) at the definite refractive index (RI) [2]. Changing the incidence angle changes the outcoming light until it reaches a critical angle. This phenomenon is called total internal reflection (TIR). When the frequency of the incident light is equal to the resonance frequency of the metal, it occurs an energy transfer from the photon of the light to the surface electrons of the metal. As consequence, the electrons move and generate an electrical wave (200 nm deep) called plasmon [3]. The surface plasmon resonance phenomenon takes place at a defined frequency of the light/angle of incidence, and it depends on the RI close to the metal surface that changes with the mass on the chip surface. The binding of molecules, within the range of the electric field, changes the mass on the chip surface and it perturbs the plasmon changing the resonance wavelength. The most widely used SPR detection method was based on the Kretschmann—Raether attenuated total reflection (ATR) configuration (see Figure 3). By the Kretschmann configuration, the dielectric constant changes of the medium near a metal film’s surface were detected by measuring the intensity changes of the reflected beam. Changing the geometry configurations, the light wavelength, and sensor surface, several SPR hybrid methods were designed, such as electrochemical surface-plasmon resonance (EC-SPR), localized surface plasmon resonance (LSPR), and SPR imaging (SPRi). In EC-SPR, a thin metal film is placed on the substrate to stimulate surface plasmons. It operates as an electrode for electrochemical detection, by providing information about the electrochemical and optical properties of the films [4,5,6,7]. Electrochemical configuration, in combination with SPR, can be used to study the kinetic reactions of biomolecules in the presence of electric fields.

Recently, EC-SPR has evolved in another hybrid technique, the SPR scanning electrochemical microscopy (SECM) [8,9]. SPR-SECM combines the sensitivity and resolution of SPR with the measure of the local electrochemical behavior of liquid/solid, liquid/gas, and liquid/liquid interfaces. The electrochemical signals are acquired using a precise ultramicroelectrode tip that scans the substrate region of interest. Changes in the recorded current depend on the distance between the electrode tip and substrate surface. This approach allows us to obtain the image of surfaces with information of topology and reactivity through moving the tip across surfaces. In LSPR is utilized a surface composed of nanomaterial with a dimension smaller than the wavelength of light.

The refractive index changes are induced by the size and the shape of the metal nanostructure and they can be used to monitor molecular binding events [10,11,12]. Controlling the size and shape of nanoparticles and the dielectric constant of the substrate, it is possible to modify and tune the LSPR characteristics [13,14,15,16,17,18,19,20]. Combining the dark-field (optical scattering) microscopy with the LSPR it is possible to evaluate local changes in the refractive index due to molecule binding events. The wavelength scanning (wavelength-shift measurement) approach is typically used to evaluate the absorption, scattered, or transmitted intensity from immobilized nanoparticles [21,22,23]. The nanoparticles size determines a highly confined electromagnetic field, and define the LSPR technique as sensitive to a single molecule. In fact, smaller nanoparticles represent an advantage for the detection of single molecules in bio-sensing approaches.

Gold and silver nanoparticles (NPs) exhibit LSPR at visible as well as near-infrared frequencies, with sharp peaks in their spectral absorbance. The absorption wavelength of the LSP is characteristic of the type of material and it is strongly dependent on the dielectric environment, the size, and the shape of the NPs [24]. One major disadvantage, however, is that LSPR sensors are prone to interference because they respond not only to refractive index variations but also to non-specific binding events. These interactions can severely compromise the measurements when working in complex matrices, and hence they limit the applicability and impact of their utilization [25]. Surface plasmon resonance microscopy (SPRM), also called surface SPRi, is a label-free method that combines the surface plasmon resonance of metallic surfaces with imaging of the metallic surface. It is an advanced version of classical SPR analysis, where the sample is monitored without a label through the use of a CCD camera. The heterogeneity of the refractive index of the metallic surface imparts high contrast images, caused by the shift in the resonance angle. SPRM can achieve a sub-nanometers thickness sensitivity and lateral resolution achieves values of micrometers scale. SPRM measurements can be made in real-time, such as measuring binding kinetics of membrane proteins in single cells, or DNA hybridization. The main advantage of SPRi technology with the use of a CCD camera is the simultaneously recording of sensograms and SPR images for the analysis of hundreds of interactions. To increase the throughput of standard SPR biosensors, Rothenhausler and Knoll [26,27,28,29] developed the SPR microscopy or imaging method. This approach suffered from a reduced sensitivity compared to conventional SPR. The SPRi additional value is to offer the opportunity to visualize a whole biochip via a CCD camera. The biochips are prepared in an array format where each spot simultaneously provides a quantity of biological information. The CCD camera provides images in real-time from hundreds of spots. The acquired images show local changes on the chip surface and provide detailed information on molecular interactions and kinetic processes.

Another approach called high-resolution SPR imaging combines the CCD camera resolution with an inverted optical microscope, equipped with a high numerical aperture oil immersion objective [30,31,32]. This configuration permits a pixel-by-pixel tracking of the reflectivity in the SPR images. Each of these pixels accordingly produces an SPR curve and the image is framed using the SPR minimum angle information.

All mentioned technologies are widely applied in biosensor application, and in the next paragraph, we will describe the applications of the emergent SPR biosensor in food, environment, security, and health (Table 1).

### 2.1. Food

One of the most common applications of SPR biosensors is in food safety, where biosensors have been developed for several classes of contaminants as foodborne pathogens, mycotoxins, plant and bio-marine toxins, toxic chemicals (mostly of anthropogenic source), preservatives, and anti-oxidants (Table 1).

For pathogen detection, different SPR biosensors have been developed. For example, Wei et al. [33] used the SPREETATM SPR system (Texas Instruments) to detect *Campylobacter jejuni* using polyclonal antibody immobilized directly on the sensor surface. The assay showed a sensitivity of 1 × 10^3^ CFU/mL.

Barlen et al. [34] used an SPR device to detect *Salmonella typhimurium* (2.5 × 10^5^ CFU/mL) and *S. enteritidis* (2.5 × 10^8^ CFU/mL). Oh et al. [35] developed an SPR-based protein chip with immobilized monoclonal antibodies against *S. typhimurium*, *E. coli* O157:H7, *Yersinia enterocolitica,* and *Legionella pneumophila*. Hearty et al. [36] produced a murine monoclonal antibody against the surface-located *L. monocytogenes* internalin A (InA). The obtained LoD was of 1 × 10^7^ CFU/mL.

Koubová et al. [37] designed a home-made device that was able to detect 1 × 10^6^ CFU/mL of *L. monocytogenes* and *S. enteritidis*. Taylor et al. [38] created an eight-channel SPR sensor that allowed the simultaneal detection of *E. coli* O157:H7 (1.4 × 10^4^ CFU/mL), *L. monocytogenes* (3.5 × 10^3^ CFU/mL), *C. ejuni* (1.1 × 10^5^ CFU/mL), and *S. choleraesuis* (4.4 × 10^4^ CFU/mL).

SPR was successfully used for the detection of small molecules such as bacterial and dinoflagellate toxins, mycotoxins, and plant toxins. An indirect test to detect aflatoxin B_1_ is also reported [39]. The produced SPR immunosensor allowed us to detect the presence of fumonisin B_1_ in milk samples with a LoD of 50 ng/mL [40].

Naimushin et al. [41] designed an SPR platform to detect the presence of sub-nanomolar concentrations of enterotoxin B, produced by *Staphylococcus aureus* in milk, seawater, and mushrooms.

Ricin represents one of the most potent plant toxins. Its detection was performed by many methods, but not with SPR until recently. Feltis et al. [42] developed a homemade biosensor to detect ricin at low concentration in respect to the minimum lethal dose (200 ng/mL).

Abrin, is a highly potent and lethal type II ribosome-inactivating toxin from *Abrus precatorius*. Its structure is similar to the structure of ricin and it has the same biochemical mechanism of action. It was developed a very sensitive assay (75 ng/mL) through the production of two human monoclonal antibodies, able to bind this toxin with high affinity and specificity [43]. Taylor et al. [44] reported the quantitative antibody-based detection of tetrodotoxin (TTX) through an inhibition assay using an SPR sensor. The assay was based on the use of anti-TTX antibody sensing surface and it allowed a detection limit of 0.3 ng/mL.

In recent years, there has been an increase in the use of tylosin in apiculture due to resistance to oxytetracycline. Caldow et al. [45] reported an SPR assay to detect the presence of tylosin with a detection limit of 2.5 μg/kg. In addition, an interesting approach for the detection of phenol employing living cells was presented by Choi et al. [46]. They fabricated a sensor surface containing the *E. coli* O157:H7 strain. The cellular damage associated to the phenol presence induced a change of SPR signal. The detection limit of phenol was 5 ppm [46]. Ascorbic acid is a commonly available nutrient which has anti-oxidizing properties. It is largely used in the industrial food processing as a preservative. Excess of ascorbic acid in food produces gastric problems. A polyaniline molecular imprinting polymers (PANI) MIP-based fiber optic sensor exploiting the principle of SPR was reported (LoD of 1.28 × 10^−^^10^ M) [47].

### 2.2. Enviroment

The SPR technique is largely applied for the detection pesticides, herbicides, aromatic compounds, chemical mixtures, and toxic metal ions that are responsible for environmental contaminations (Table 1).

One of the most widespread classes of pollutants is acetylcholinesterase inhibitors pesticides (organophosphate and carbamate) widely used for pest and insect control in agriculture, livestock, and domestic uses.

Several SPR optical biosensors have been developed to detect acetylcholinesterase (AChE) inhibitors. However, the small size of the inhibitors produces a low shift in resonance and the consequentially a poor sensitivity. To overcome this problem in the SPR assays are applied nanoparticles (NPs) that promote a significant shift in the angle of plasmon resonance.

Chlorpyrifos (CPF) is one of the most diffuse pesticides, and Yao et al. [48] reported an innovative detection method based on the synthesis of magnetic MIP-NPs. MIPs present recognition sites for CPF. NPs were synthesized using Fe_3_O_4_. The Fe_3_O_4_-NPs showed a high molecular weight and magnetic features. Integrating the CPF-imprinted Fe_3_O_4_ NPs to an SPR chip resulted in a significant signal amplification due to the high molecular weight of NPs. The SPR biosensor showed a detection limit for CPF of 0.76 nM.

Atrazine, a member of the triazine class, is an herbicide. It is used for the control of the broadleaf weeds in crops [49]. Due to its toxic nature, it can affect the ecosystem and human health causing cancer or reproductive abnormalities. The monitoring of this molecule in the environment (air and water samples) is of fundamental interest. Agrawal et al. developed a method for the detection of atrazine by coupling the molecular imprinting technology (MIT) with the SPR approach over the use of an optical fiber substrate [50]. The MIPS, able to recognize the atrazine, were immobilized onto a fiber optic substrate. The developed SPR sensor showed to be very sensitive (LoD of 1.92 × 10^−14^ M).

Monitoring the presence of harmful chemicals such as benzene, toluene, ethylbenzene, xylene, and volatile organic compounds (VOCs), is an interesting and emerging field of SPR applications. In fact, SPRi may provide comprehensive information on the composition of VOCs besides a simple detection. The integration of SPRi to the micro-gas chromatography system allows for a simultaneous separation and multidimensional detection of target chemicals in a gas mixture. Brenet et al. [51] have developed an SPRi chip for VOCs sensing in the gas phase. The developed sensor showed high selectivity and the capability to discriminate between different VOCs differing only for a single carbon atom.

Metal ions contaminations represent still a serious problem for the environment and health. The exposure to metal ions can cause harm and affect human health. The coupling of graphene oxide (GO) nanoparticles with SPR method improved the detection capabilities of toxic metal ions. Lokman et al. [52] developed an SPR sensor for the detection of Pb^2+^ with high sensitivity 0.77 ppm^−1^. They enhanced the sensitivity of the SPR sensor by developing a gold-chitosan-graphene oxide (Au/CS/GO) nanostructured thin film [53]. To detect Co^2+^ Saleviter et al. [54] prepared an active layer immobilized 4-(2-pyridylazo) resorcinol in a chitosan–graphene oxide composite (PAR-Cs-GO). The obtained sensor was able to detect Co^2+^ as low as 10 ppb. Daniyal et al. [55] developed an SPR sensor to detect Cu^2+^ with an LoD of 0.01 ppm. They prepared a sensor surface altering the nanocrystalline cellulose by hexadecyltrimethylammonium bromide and GO composite (CTA-NCC/GO).

### 2.3. Security

The SPR methodology is deeply applied also in the field of human security. In fact, SPR based devices able to sense and detect explosives such as the trinitrotoluene (TNT) and/or chemical warfare agents (CWAs) such as the lachrymators (like the capsaicin, have been developed in the last decade (Table 1).

The most diffused SPR approach is the immunosensor because the antibodies have the capability of detecting low molecular weight compounds like 2,4,6-TNT and capsaicin.

The detection of TNT by an SPR immunosensor was reported by Zeck et al. [56], using an indirect competitive assay. The surface sensor was immobilized a 2,4,6-trinitrophenyl-keyhole limpet hemocyanin conjugate. As detection molecules, commercially available monoclonal antibodies against 2,4,6-TNT were utilized [57]. Another approach to detect TNT used a competitive immunoassay based on a dendrimer-modified SPR surface [58]. A thiol SAM combined with a poly(amidoamine) (PAMAM) dendrimer provided the support structure for attachment of dinitrotoluene-keyhole limpet hemocyanin conjugate (immobilized antigen). Using a monoclonal antibody as a detection molecule, a LoD of 110 pg/mL was achieved [58].

Combining fiber optic SPR and MIP technique, Cennamo et al. [59] developed a highly sensitive TNT SPR sensor. The fiber optic surface was realized by the coating of 60 nm thick gold film over the core of the fiber, the MIP was immobilized on the gold surface. The developed sensor showed a LoD of 5.1 × 10^−5^ M. Using an SPR immunosensor approach Onodera et al. [60] detected the capsaicinoids. To recognize a vanillyl group of capsaicinoids a polyclonal antibody against homovanilic acid (CCH) was developed. An indirect competitive assay was performed by immobilizing the capsaicin analogs via a SAM on the surface of the sensor. Different capsaicinoids, homovanillic acid, and vanillylamine (4-hydroxy-3-methoxybenzylamine) were used for the sensor chip on which vanillylamine was immobilized. The developed indirect competitive assay shows an LoD of 150 ppb [61].

### 2.4. Health

The application of new methods of analysis in the health field is characterized by the typology of the analytes and by the heterogeneity of the sample (matrix) to analyze. SPR is an exciting tool for health diagnosis and clinical treatment monitoring. In fact, SPR biosensors were developed for the detection of small molecules like drugs (steroid hormones, cocaine, ecstasy, heroin, amphetamine, antibiotic, sulfamethazine, vitamin, nicotine, melamine, erythromycin, and dopamine), polypeptides, proteins (growth factors, cancer biomarkers, antibodies, and serum proteins), DNA molecules and whole organisms (bacteria and virus) (Table 1). In particular, SPR-immune biosensors have been largely applied to identify biomolecules of interest, taking advantages from the large availability of specific antibodies from the marketplace and the simplicity to produce ad hoc antibody.

The capability of SPR to analyze several types of biological fluids and tissue matrices (saliva, blood, whole cell, and etc.) and the possibility to monitor in real-time the association-dissociation process of biological molecules, have prompt the development of SPR applications for in vivo assays. The application of SPR for in vivo analysis has permitted us to clarify several molecular aspects of cellular functioning.

The detection of pathogenic viral agents takes advantage of the highly sensitive and selective SPR methods. In particular, analytical methods based on PSPR or LSPR are widely utilized. The detection of the Newcastle disease virus (NDV) was possible by an LSPR immunosensor developed by Luo et al. [62]. They coated excessively tilted fiber grating (Ex-TFG) with AuNP, and monitoring the resonance wavelength shift, achieved an LoD of ~25 pg/mL.

The HIV virus-like particle detection was achieved by an immunosensor based on the LSPR mechanism [63]. The diagnosis of dengue viral infection was possible by a rapid propagating surface plasmon resonance (PSPR)-based immunoassay, where a neutravidin-biotin monoclonal antibody (the sensing element) was immobilized on a thin gold film. An LoD of 2 × 10^4^ particles/mL was obtained [64]. Avian influenza A H7N9 was detected by Chang et al. [65]. They developed an intensity-modulated surface plasmon resonance (IM-SPR)-based immunosensor. The observed LoD, in samples spiked, was 402 copies/mL.

For the family of steroid hormones, estradiol and progesterone were detected by SPR with online in-tube solid-phase microextraction (SPME) system, to monitor estrogenic cycles in cows, with an LoD of 3.5 ng/mL [66] for the progesterone and an LoD of 170 pg/mL for the estradiol [67].

The detections of cortisol and testosterone in human saliva are of great interest because they are associated with hormonal disorders such as Addison’s disease and Cushing’s syndrome. Coupling the covalent immobilization of antibodies and OEG linker technology was possible to construct highly sensitive SPR immunoassays for both cortisol [68] and testosterone [69]. The LoD values obtained were 49 pg/mL and 15.4 pg/mL for cortisol and testosterone respectively.

The development of a high-throughput detection assay allowed the analysis of the bile that is a complex fluid because contains many different analytes of interest. Sulfamethazine and sulfadiazine were detected [70]. The same technology was, also applied for measurements of clenbuterol and ethinylestradiol in urine and sulfamethazine, sulfadiazine, and enrofloxacin in milk [71].

Another class of analytes is represented by antibiotics used for prevention and treatment of several bacterial infections. Large amounts of antibiotics, however, may harm the human body via allergic symptoms and other diseases. Tetracycline (TC) and oxytetracycline (OTC) are two very diffuse antibiotics. A detection method coupling MIT and fiber optic SPR technique was developed coating an Ag thin film over the core of the optical fiber followed by a MIP TC/OTC layer. The sensor operation was checked for the tetracycline concentration range 0–0.96 µM and for the OTC concentration range 0–0.96 µM [72].

Shrivastav et al. [73] improved the sensitivity (an LoD of 2.2 × 10^−9^ M) of the TC sensor by incorporating the combined phenomenon of SPR and LSPR. The sensor was fabricated by including the Ag nanoparticle layer between Ag and MIP-TC layer [74].

Erythromycin (ERY) is another diffuse antibiotic used to reduce the activities of Gram-positive and Gram-negative bacteria. Its wide use results in its presence in foodstuffs and derivatives. The detection of ERY in an aqueous medium was allowed by an SPR sensor developed using ERY-MIP nanoparticles. The sensor was able to sense an ERY concentration range from 0.0 to 50 µM [74].

The SPR approach is also used for vitamin detection. The most important vitamin is vitamin B_3_, also known as niacin/3-pyridinecarboxamide, essential for maintaining healthy skin, proper breathing, and metabolism and to keep the nervous system fully functional. A molecular imprinted hydrogel-based SPR fiber optic sensor utilizing colloidal crystal templating was reported to detect the vitamin B_3_ (analyte concentration of 0 to 10 mg/mL) [75]. A similar approach was developed for riboflavin/vitamin B_2_, with a concentration range of 0–320 µg/mL [76].

Another interesting class of analytes is represented by drugs like cocaine, nicotine, ecstasy, heroin, and amphetamine. For drug detection, the most diffuse SPR method is the LSPR that uses a combination of antibodies and antigen-protein conjugates immobilized on the array [77].

Nicotine is reported to affect the nervous system which can result in paralysis and respiratory block. The detection of nicotine in human body fluid was performed by Cennamo et al. [78] using a fiber optic L-nicotine sensor. They coupled SPR and MIP on tapered PMMA plastic fiber. The sensor showed a response time of 10 min and an LoD of 1.86 pM [78].

Another application of SPR technology is the diagnostic screening of serum samples, epitope mapping, and protein expression profiling. Nagel et al. [79] restricted their SPR studies for serological detection of *Lyme borrelioses* to two widely used antigens. The whole proteins as well as two peptides, representing immunodominant domains, were used as capture probes. De Boer et al. [80] used an SPR platform that combines the microarray principle with SPR detection in one flow chamber. The microarray contained 144 different glycans derived from the human parasite *Shistosoma mansoni* and was used for the simultaneous detection of glycan-specific serum antibodies.

An SPR biosensor was used to detect antibodies directly from human blood serum against the immunoreactive peptide epitope of Epstain-Barr Virus (EBV) nuclear antigen. The detection limit was estimated to be 0.1 ng/mL, which is lower by an order of magnitude than the detection limit of Enzyme-Linked Immunosorbent Assay (ELISA) [81].

The serum components present in low concentration, like IgE or cytokines, may not be detected, but Battaglia et al. [82] demonstrated the detection of biologically relevant levels of the cytokine IL6 in cell culture media using an SPR sensor. To reduce the non-specific protein adsorption, the sensor surface was modified by a layer of NHS ester and 16-mercaptohexadecanoic. Weinhart et al. [83] suggested SAMs of linear polyglycerol derivates for gold surfaces.

The ability to detect biomarkers in blood samples is really important for clinical applications. However, biomarkers in blood samples are present in small concentrations. The SPR method, with the aid of nanoparticles, represents an interesting tool to overcome this issue. NPs-SPR sensors were developed for detection of prostate-specific antigen (tPSA) [84], carbohydrate antigen 15-3 (CA15-3) [85], carcinoembryonic antigen (CEA) [86], C-reactive protein (CRP) [87], human epidermal growth factor receptor 2 (HER2) [88], estrogenic receptor (ER) [89], progesterone receptor (PR) [90]. By using 40 nm nanoparticles conjugated with the PSA antibody, a tPSA assay was performed on 75% human serum at a detection limit of 0.29 ng/mL^−1^ (8.5 pM). C-reactive protein (CRP) is a principal blood serum biomarker for conventional inflammation, Jung et al. [87] developed a spectral SPR system to detect C-reactive protein (CRP) in human sera immobilizing the CRP monoclonal antibody to dextran functionalized gold surface.

An important biomarker for malignant tumor progression and metastasis is the human matrix metalloproteinases-9 (MMP-9). An SPR-based immunosensors for real-time and label-free detection of recombinant MMP-9 was reported by Mohseni et al. [91]. Combining the surface hybridization, surface ligation, and nanoparticle amplification for single-nucleotide polymorphism (SNP) genotyping in BRCA1 gene Li et al. [92] developed an SPR method to evaluate the presence of a single mismatch on BRCA1 gene by using nanoparticles with oligonucleotides complementary to the ligation probe DNA. They were able to detect the SNP at concentrations as low as 1 pM. The nanoparticles substantially helped to overcome the limitation of conventional SPR biosensors [93].

Additional interesting area of health is the in vivo monitoring of physiological phenomena such as cellular response, cell adhesion, and cellular products, as well as detection of cancer cells and bacterial cells. Since cells respond to stimulation of reactive molecules, the cell-molecule interaction cause changes in the SPR signal, and, of consequence, SPRi represents a suitable technology to reveal cell-molecules interactions [94,95,96].

Yanase et al. [96], developed an SPR sensor to detect the presence of intracellular events observed the changes in the size of the cell adhesion area. It has been observed that the value of RI near the plasma membrane, which could be determined by the accumulation and rearrangement of the proteins activated by the transduction of the intracellular signal, changes profoundly following exogenous stimuli.

The precise mechanism for cells to determine such large variations of RI is not yet fully understood. However, detections and/or analyses of cellular functions were studied by measuring the value of RI with respect to real-time adhesion and morphological changes in cells in response to various agents [97]. For example, the use of an infrared SPR sensor based on FTIR-SPR with Fourier transform has made it possible to know the changes in the biochemical composition of the membrane, such as cholesterol [98,99]. An SPR sensor with cells that express the olfactory receptor has been designed for the detection of volatile compounds [98,99,100], to detect the reactions of cancer cells against an anticancer drug [101,102] or small morphological changes that occur following the induction of apoptosis in cells [103].

## 3. Fluorescence-Based Biosensors

Fluorescence is an optical phenomenon characterized by the absorption of photons at one wavelength followed by emission at a longer wavelength. In the fluorescence process, described by a typical Jablonski diagram, vibrational relaxation results in a loss in energy between the absorbed and emitted photons (Stokes shift) [104]. Fluorescence spectroscopy is a very efficient technique for biomolecules detection, and fluorescence-based methods are the most commonly used in the field of optical biosensors. This methodology combines the high sensitivity of fluorescence detection, with the high selectivity provided by specific MREs such as ligand-binding proteins, antibodies, aptamers, and/or through the use of fluorescent nanoparticles (quantum dots, metal nanoparticle, etc.). The growing availability of fluorescent molecules (including fluorescence proteins, small molecules dyes and nanoparticles), the development of a variety of strategies for biosensors design joined to the great advances in analytical platforms (fluorescence spectroscopy for solution-based assays, microplate readers, microscopy for cell imaging, flow cytometry, in vivo imaging techniques), have allowed the development of a variety of sensors for both in vitro and in vivo analysis.

**Figure 4:**
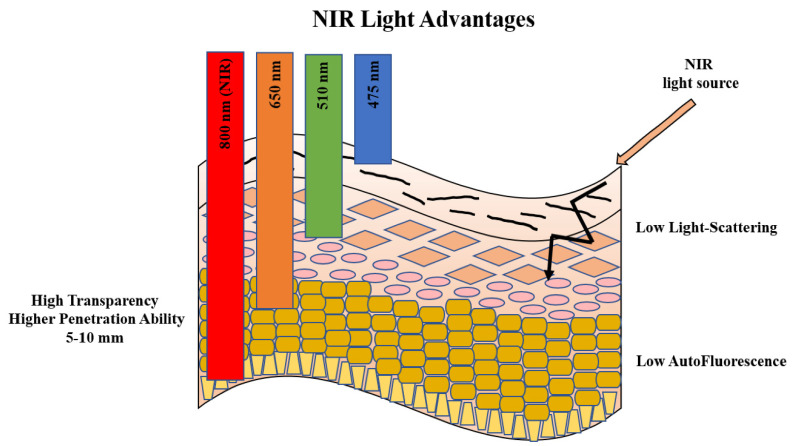
Advantages of the use of near-infrared light. The fluorescence imaging technique with near-infrared is widely used due to its superior qualities since their range of absorbance and fluorescence corresponds to the region of highest transparency of biological tissues. In fact, at these wavelengths, the light penetrates deeper through animal tissue (because the combined effects of tissue absorbance and light scattering are at a minimum), and autofluorescence of biological tissues is less pronounced compared to the visible range.

In particular, in the last years the production of near-infrared (NIR) dyes and proteins (NIR-FPs), has given a new impulse to the research, since their range of absorbance and fluorescence (~650–900 nm) corresponds to the region of highest transparency of biological molecules [105]. Because biomolecules have very low absorption, reduced light scattering, and lower autofluorescence in the NIR region, the use of NIR fluorescent dyes reduces significantly the background signal due to the matrices (in vitro analysis), and to tissues (in vivo imaging). Moreover, NIR-sensors can be used in combination with visible probes and/or proteins, expanding the possibilities of multicolor imaging (see Figure 4).

The fluorescence-based detection offers a large number of subclasses based on different optical principles, including fluorescence intensity (FI or steady-state), Forster resonance energy transfer (FRET), fluorescence polarization (FP), and fluorescence correlation spectroscopy (FCS).

Fluorescence steady-state (FI) is based on the direct measurements of fluorescence emission of specific molecules as a consequence of excitation by a light source. The obtained data are shown as an emission spectrum, reporting fluorescence intensity as a function of light wavelength [104].

FRET is a physical process in which the energy is transferred from an excited molecular fluorophore (the donor) to another fluorophore (the acceptor). It is dependent on the distance between the donor-acceptor pair. In particular, the efficiency of FRET process is dependent on the inverse sixth power of intermolecular separation. If the donor and the acceptor molecules are positioned within the Förster radius, typically 3–6 nm, the efficiency is high [104,106,107]. This technique is widely used to investigate a variety of biological phenomena that produce changes in molecular proximity such as ligand-protein interaction, protein-protein interaction, etc. [108].

The FP method is used to study the protein-ligand and protein-protein interactions and it is based on the principle that when a molecule is excited by a plane-polarized light, the polarized emission is dependent on the lifetime of the excited state compared with the rotational time motion (Brownian motion in solution). At constant temperature and viscosity values, the FP value will be directly dependent on the effective molecular size of the excited molecule. Consequently, the use of the FP method allows us to detect interaction between the MRE and specific analytes. In the case of small molecules (that have fast Brownian rotation in solution) the FP values are low, whereas for larger molecules (e.g., in complex with antibody) the FP values are higher [104].

In FCS analysis, the fluctuation of fluorescence intensity in the time is measured. The fluctuations of labeled molecules can be due to photophysical properties of the label or movement of the molecule and its diffusion time, as a consequence of the size and the shape of molecules. FCS technique is used for different applications in biology, biophysics, and chemistry such as diffusion. In this case, it can be used to monitor Brownian diffusion [109], anomalous diffusion [110,111,112], flow [113,114], conformational changes, molecular binding, and/or chemical reactions, at single-molecule sensitivity. The FCS technique is applied mainly in solution but attempts to apply it for in vivo analysis are also reported in literature [115]. All of these techniques have been employed as output for fluorescence-based biosensors and in the next paragraphs, we will describe the state of the art and the recent results in the implementation of fluorescence-based sensors for monitoring specific analytes for food, environment, health, and security fields (Table 2).

### 3.1. Food

Due to the increase of the world population and the need to improve the effectiveness and efficiency of food chains [116], in recent years, an increasing number of biosensor technologies applied to food safety have been developed. These approaches allow to monitor the quality chains from food farming, production, process, packaging, transportation, to all the consuming ways [117]. The latest developments in optical biosensors to detect the presence of toxins, antibiotics, hormones, and food allergens, are hereafter described (Table 2).

Globally, antibiotics have been widely used in animal husbandry for over 50 years with the principal aim of preventing and/or treating diseases affecting animals. Currently, the main compounds used in food belong to the family of β-lactams, which includes penicillin G (PenG), and cephalosporins, both of which are used extensively as food additives in the treatment of livestock. In literature are reported different optical biosensor for monitoring antibiotic residues in different foodstuff [118] such as milk. For the detection of antibiotic residues in food, the publications date from 1985 to 1997 [119,120,121]. Different methods have been developed using classical fluorescence for the detection of antibiotic residues such as aminoglycosides in milk [122] and tap water [123], or sulfadimethoxine in milk [124]. Other screening methods for veterinary drug residues were developed through time-resolved fluorescence-immunoassay (TR-FIA) between 2006 and 2015 [125,126,127,128,129,130,131,132]. These methods allow us to detect the presence of a single antibiotic (e.g., chloramphenicol) or a group of antibiotics (e.g., fluoroquinolones). Biosensors based on fluorescence detection are compatible with multiplexing technology. A multiplex biosensor based on fluorescence was developed by Chen et al. [133] for the detection of eight antibiotics in chicken and porcine muscle and liver. A microarray of different spots was printed on a modified glass chip. Recently, it has been developed a fluorescence polarization method to detect directly in milk the presence of PenG [134] and Ciprofloxacin [135]. This methodology is based on the increase of fluorescence polarization emission of a fluorescence-labeled compound derivative upon their binding to specific antibodies. The competition between the milk unlabeled contaminant and the fluorescence-labeled contaminant allows us to detect the presence of the target compound in milk. The results obtained suggest that the method could be applied directly in milk without interference. For the PenG detection, the LoD of the method was 1.0 nmol/L, which is much less than the required maximum residual limit (MRL) in EU regulations (12.0 nmol/L) [134] while the obtained results for the ciprofloxacin reached a sensitivity of 1 ppb [135], 100 times lower than MRL of ciprofloxacin in milk, as fixed by the European Union regulation (100 ppb).

Mycotoxins, as patulin (PAT), are toxic secondary metabolites produced from different fungal species belonging to the genera *Penicillium*, *Aspergillus*, and *Byssochlamys*. These fungi can grow on a large variety of food, including fruits, grains, and cheese. In the case of PAT, its presence in apple products is a crucial issue because it is the measure of the product quality. In the work of Pennacchio et al. [136], it has been developed a fluorescence polarization approach based on the use of NIR fluorescence probes. The innovation of this approach is the use of these fluorophores coupled to anti-PAT antibodies. It makes possible the detection of PAT directly in apple juice samples without any pretreatment. The LoD of the method was 0.06 μg/L, a value that is lower than MRL of PAT fixed at 50 μg/L from European Union regulation [136].

An FCS-based assay to detect traces of ochratoxin A in wine is also reported in literature [137]. This assay combines the use of high-avidity IgG antibodies with the sensitivity of the single molecules detection instrumentation to detect the presence of 0.0078 ng ochratoxin A.

Different works showing the application of innovative methods for point-of-care detection of food contaminants have also been reported [138]. In the case of mycotoxins detection, recently it has been developed a user-friendly approach, based on the use of the modified commercial glucometer to detect aflatoxin M1 (AFM1) in whole milk [139]. The assay allows to detect in less than 2 h the presence of toxin without any pre-concentration and/or pre-treatment of milk. The novelty of the method is that the presence of AFM1 is correlated to the glucose concentration produced by an invertase-conjugated anti-AFM1 antibody. The produced glucose is detected by the glucometer. The assay is sensitive since it is possible to detect the presence of 27 parts per trillion (ppt) of AFM1 in whole milk, a value lower than the AFM1 quantities, in milk and dairy products, set by the European Union regulation (50 ppt).

Steroids are a class of hormones improperly used in livestock as growth-promoting agents. Due to their high risk for human health, the European Union has strictly forbidden the addition of all-natural and synthetic steroid hormones to food-producing animals.

Recently, an FP assay, based on the use of Fourier-transform infrared (FT-IR) fluorescent probe, was developed to detect 17β-estradiol directly in milk samples. This method displays LoD values of 10 pmol [140].

An FP assay based on estrogen receptor α-ligand binding domain (ER-LBD) to monitor stilbene estrogens (hexestrol, dienestrol, and diethylstilbestrol) in milk, has been recently developed. This method displays LoD values of 2.94 nM, 2.89 nM, and 3.12 nM for hexestrol, dienestrol and diethylstilbestrol, respectively [141].

Allergic individuals are exposed to food allergies such as cow milk allergy (CMA). The casein fractions (S1-casein, αS2-casein, β-casein (28%), and κ-casein) and whey proteins (α-lactalbumin β-lactoglobulin, bovine albumin serum (BSA), lactoferrin, and immunoglobulins) are the cow’s milk allergenic proteins. In literature, different biosensor solutions are reported in order to detect these proteins. A fluorescence immunoassay in which a monoclonal antibody against α-lactalbumins is covalently conjugated with the CdSe/ZnS quantum dots (QDs) using crossing-linking reagents. The obtained competitive fluorescence-linked immunosorbent assay (FLISA) method exhibited high sensitivity with an LoD value of 0.1 ng/mL [142].

A specific novel type of bivalent apta-sensor based on silver-enhanced FP for detection of lactoferrin (Lac) in milk powder was developed [143]. This method utilizes a specific aptamer, produced by SELEX (systematic evolution of ligands by exponential enrichment) method, and modified with the addition of a linked with signal-molecule fluorescein isothiocyanate (FITC) through enhancer silver decahedral nanoparticles (Ag10NPs). This configuration proved a sensitivity assay with a detection limit of 1.25 pM. Additionally, the FCS technique was used to develop a competitive assay for the detection of the presence of food allergens such as gluten in food for celiac patients [144].

### 3.2. Environment

For environment control, biosensors are often used instead of the traditional instrumental analyses for detection and quantification of specific pollutants. In particular, in this section, we report the application of nanoparticles-based biosensors for the detection of important environmental contaminants, such as heavy metal, hormones, pesticides, and aromatic compounds (Table 2).

In the case of detection of the presence of heavy metal in water, some approaches based on fluorescence spectroscopy methods are reported in literature [144,145]. In these assays, the methallotionein and its derivative (a peptide that mimics its binding site) were used as a probe for cadmium and other heavy metal detection in water. Recently, the green fluorescence protein (eGFP) was also used for sensing the presence of heavy metal in water. This approach uses the combination of two fragments of split-eGFP that form a native structure with an inserting metal-binding loops (MBLs) between β-strands 9 and 10 of the eGFP. The variation of the registered fluorescence emission is a consequence of the conformational changes upon interaction between MBLs and target analytes [146].

Due to their effect on endocrine system, some articles in literature report the application of biosensors for monitoring the presence of hormones in the environment. An example is represented by the total internal reflectance fluorescence (TIRF)-based biosensor for detection of steroid hormone testosterone [147]. In this work, the authors used a specific monoclonal antibody against testosterone. They created the biosensor assay to determine the presence of the analyte in water with a detection limit of 0.2 ng/L.

Nanoparticles have been largely used in environmental analysis. In particular, semiconductor QDs are the most commonly used in fluorescence sensing approaches, due to their main characteristics such as higher brightness, reduced photobleaching, and long lifetimes. Recently, many research groups have described the applications of QDs-based fluorescence assays to detect the AChE activity and organophosphorus pesticides [148,149,150,151,152,153]. Yi et al. [154] discovered that the fluorescence intensity of label-free silicon quantum dots (SiQDs) is affected by enzyme-generated H_2_O_2_. They developed a SiQDs-based sensor for pesticide detection based on the fluorescence emission quenching of SiQDs induced by H_2_O_2_ as a consequence of the hydrolyses of acetylcholine to choline [155]. This method was used to detect carbaryl, parathion, diazinon, and phorate at low concentrations 7.25 ng/L, 32.5 ng/L, 67.6 ng/L, and 0.19 mg/L, respectively. In the presence of pesticides, the activity of AChE was inhibited with the reduction of H_2_O_2_ and an increase of the SiQDs fluorescence.

Metal (e.g., Au or Ag) and nanoclusters (NCs) are an emerging technology that offers a compromise between the photostability and brightness of quantum dots and the flexibility of fluorophore dye [156]. This technology was used for chemical and biological detections and cellular imaging applications. For example, Li et al. [157] developed a fluorescence sensor for AChE based on the use of thiocholine-induced fluorescence quenching of DNA-templated copper/silver nanoclusters (DNA-Cu/AgNCs). The obtained sensor allowed us to detect amounts of 0.05 mU/mL. Li et al. [157] also synthesized the denatured BSA-protected AuNCs and demonstrated its application for fluorescence detection of AChE activity directly in human serum. In particular, the fluorescence emission of AuNCs was quenched by the produced thiocholine due to the combination of thiocholine with the dBSA-AuNCs. The method showed a detection limit of 0.02 mU/mL. Zhang et al. [158] used BSA-stabilized gold nanoclusters (BSA-AuNCs) as fluorescence reaction substrate to detect the activity and phosphorylation of AChE and dimethyl-di-chlorovinyl phosphate (DDVP). Using this method, the DDVP was determined with a detection limit of 13.67 pM.

Carbon monoxide, sulfur and nitrogen oxides, particulates and volatile organic compounds (VOCs), derived from various combustion processes are the main contaminates detected, due to their strong impact on human health. A group of VOCs called BTEX, (benzene, toluene, ethyl-benzene and xylene isomers), generally is monitored for both ambiental and industrial applications as well as health and safety claims. Benzene is the main VOC, belonging to group-I carcinogens. In literature, different approaches are reported for benzene detection based on a single-walled carbon nanotube (SWNTs) and on the use of cells such as *Pseudomonas putida* or *Escherichia coli* [159].

Recently, Capo et al. [160] have developed a protein-based FRET assay for benzene detection using an odorant-binding protein (OBP) as MRE. The assay is based on the competition between the 1-aminoanthracene and benzene to OBP. The displacement of 1-AMA, as a consequence of benzene presence, is followed as a change in the FRET signal. The detection limit of the assay was 3.9 μg/m^3^, a value lower than the European Union limit.

### 3.3. Security

Development of biosensors for detection of biological warfare agents such as bacteria, virus, and toxins are often attempted using various devices ranging from electrochemical, to optical and piezoelectric, with applications in military, health defense, and security [161] (Table 2).

Ricin A is a lectin produced by the castor bean (*Ricinus communis*) plant and is widely known for its highly toxic nature [162]. This compound is considered as a potential biological weapon and is listed as a category B bioterrorism agent by the Centers for Disease Control and Prevention in the USA [163]. In fact, documented events confirm its use in bioterrorist attacks. In literature, a detection method based on the application of gold nanoclusters (AuNCs@ew) is reported [143,164]. In this assay, the authors used AuNCs nanoparticles functionalized with chicken ovalbumin, that are able to recognize ricin. The LoD was about 400 nM.

Rapid and reliable identification of *Bacillus anthracis* is pivotal, especially in the event of suspected deliberate release of anthrax spores. With the aim to detect the presence of this bacterium polyclonal antibody against *Bacillus cereus,* a safe simulant for the *Bacillus anthracis,* are produced and used for lab-on-chip development [165].

Different methods that combine nanomaterials and polymers have been used to provide hybrid devices to monitor anthrax [166].

FRET assay, associated with cytofluorimetry methods, was developed by Stopa [167], in which the variation of FRET signal was used to detect the presence of spores of *Bacillus anthracis*. Finally, a luminescent adenylate-cyclase assay was developed to evaluate *Bacillus anthracis* edema toxin activity [168].

### 3.4. Health

Fluorescence-based sensors are nowadays ubiquitous, being spread across biomedical research and clinical practice. In this field, together with sensors for in vitro analysis, a prominent role is played by sensors able to monitor specific analytes in vivo, from living cells to the whole body, in animal models.

Fluorescence-based techniques have a prominent role to detect specific proteins for both laboratory and clinical uses, in vitro.

In numerous clinical applications, it is needed to quantify cytokines and hormones at very low concentration (up to sub-fg/mL ranges). Recently the application of nanomaterial-based approaches was demonstrated to be extremely promising to allow this sensing performance. In the following paragraphs we describe the recent applications for quantitative detection of cytokines, dopamine, and hormones (Table 2).

Several sensing methods based on a nanomaterial approach to detect cytokines are reported in the literature [169]. An example of fluorescence multiplexed cytokine detection, using polymeric beads with a size of 3.1 µm, is represented by the detection in parallel of ten different cytokines (VEGF, IP-10, IL-8, MCP-1, CCL2, TIMP-1, RANTES, MIP-1β, Eotaxin-2, and IL-6). The obtained results showed a higher sensitivity, compared to the enzyme-linked immunosorbent assay (ELISA) measurements, in the range from 8 to 469 pM. In addition, the assay was applied directly in the saliva supernatants of patients with pulmonary inflammatory diseases [170] using a similar multiplexed bead-based approach, and it was used to analyze the concentrations of 48 cytokines in the plasma, saliva, and urine [171].

Fluorescence-based protein microarray assays were developed using different substrates, including nitrocellulose, glass slides, aldehyde-modified glass, epoxy coated glass, and BSA-N-hydroxy-succinimide (BSA-NHS) coated glass. In microarrays, the signal value and the sensitivity, are largely influenced by the substrate surface. Consequently, new substrate materials for microarrays are required to overcome the issues of non-specific protein adsorption and high background to signal ratio. Teflon derivative, a fluorinated ethylene propylene (FEP) membrane, was recently developed as a fluorescence microarray substrate to reduce background signal in cytokine detection. The FEP membrane, a polydopamine micro-spot array was fabricated for protein conjugation and this structure allows to obtain LoDs for IL-1β, IL-6, and IL-10 of 8.91, 1.33, and 6.12 pg/mL, respectively [172].

The fluorescence enhancement phenomenon in the presence of noble metal nanoparticles was also used for cytokine detection. An enhanced sensitivity was obtained for the multiplexed fluorescence detection of IL-6, IFN-γ, IL-1β, and VEGF by coating the 4–8 µm glass microbeads with nanosized Au islands (~100 nm in size with 10–30 nm spacing) [173]. Using a QDs six different cytokines of TNF-α, IL-8, IL-6, MIP-1β, IL-13, and IL-1β were detected down to the pM concentrations [174].

Finally, photonic crystals (PCs), periodic nanostructures of dielectric materials, fabricated on a surface, were used for cytokine detection. Using this PC substrate, the detection SNR was increased by 5-folds by using sandwich assay format of TNF-α and a Cyanine-5 (Cy5) as label. The PC-enhanced fluorescence assay of TNF-α yielded an improved LoD of 6 pg/mL respect the same assay performed on a glass slide (18 pg/mL) [175].

QDs are much more photostable than most fluorophores. They were used in several assays to detect dopamine (DA) in addition to other fluorescent nanomaterials such as CDs, gold nanoclusters (Au NCs), Au NPs, silica NPs, polymer NPs, and CNTs. In particular, in this sensing approach, the interaction with DA with the nanomaterial changes the fluorescence intensity allowing to obtain high sensitivity of fluorescence-based sensors for DA. The lowest detection limit of 0.1 pM was achieved by using functionalized CDs with boronic acid and amino groups [176].

Different optical biosensors are reported in literature and they are becoming an alternative detection method to quantify hormones in human-derived target fluids. Recently, it has been reported a method to detect 17β-estradiol using specific short-chain oligonucleotides (aptamers) labeled with a specific extrinsic fluorescent dye. The sample was excited with a laser beam at 635 nm [177] and the mixture was pumped to the photosensitive unit. The sensing process can be completed in less than 10 min, directly in human fluid with a detection limit of 2.1 nM.

A gold nanoparticle-based fluorescence immunoassay to detect 17β-estradiol in human urine has been also reported [178]. This sensing system consists of two types of nanoparticles: magnetic microparticles (MMPs), which were functionalized with an anti-17β-estradiol antibody as a capture probe, and double codified gold nanoparticles modified with biotin and anti-rabbit antibody as a signal amplifier. In the optimized conditions, the detection limit for this system is 6.37 × 10^−6^ ng/mL.

Fluorescence biosensors are a robust method also for in vivo analysis, allowing imaging of molecules and biological processes, with high spatial and temporal resolutions. Several sensors have been reported both for fundamental and applied research, including sensors for ions (Ca^++^, Na^+^, K^+^, fluoride, Zn, Chloride, Mg^++^, Hg^++^) cell metabolites (NAD(P)H/NAD(P)^+^ ratio, ATP/ADP ratio, glucose, lactate, glutamate), reactive oxide species (hydrogen peroxide, superoxide anion radical, hydroxyl radical, peroxynitrite), reactive sulfur species, redox membrane potential, pH, transmembrane voltage, neurotransmitters, and enzymatic activities (small GTPases, proteases, kinases, phosphatases, acetylase/deacetylase). Fluorescence biosensors for in vivo imaging can be engineered with several designs [179,180], based on a single fluorescent protein/probe or on FRET pairs of fluorescent proteins/probes. Fluorescence intensity acquisition is the most commonly used as readout, compared to lifetime and anisotropy measurements, not discussed in this review.

**Figure 5:**
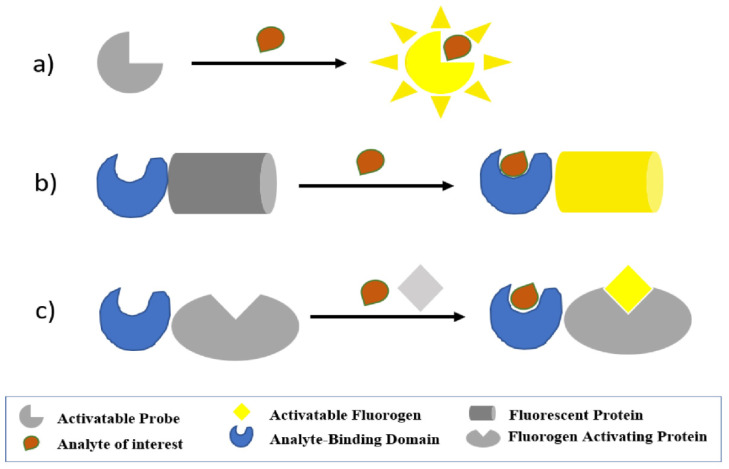
Principle of the main in vivo detection systems. (**a**) Turn on (activatable or smart) probes are exogenously added and they light on upon the binding of the analyte (hydrogen ions, ROS, calcium, ClO, etc.). (**b**) Genetically encoded sensors arise from the fusion of an analyte binding domain to a fluorescence protein; they are expressed in cells and they can change their fluorescence features when binding to ligand. (**c**) Chemical-genetic reporters consist of an analyte binding domain fused to a fluorogen-activating protein tag. These proteins are not fluorescence but they are able to activate a cognate fluorogen that in turn becomes fluorescent when exited. Sensors for ions and other molecules are designed in order to light on only when they bind the analyte.

In general, fluorescence in vivo biosensors can be mainly subdivided into endogenous genetic encoded reporters, sensors based on exogenous fluorescent agents, and hybrid systems (chemical-genetic sensors) (see Figure 5).

Genetically encoded biosensors consist basically of a chimeric protein derived from the fusion of a sensing moiety with a component providing a readout, that can be expressed and regulated intracellularly, and are passed through cell generations. The most common strategies for genetically encoded sensors are based on circularly permuted fluorescence proteins (cpFPs), bimolecular fluorescence complementation (BiFC), and FRET systems [180]. In cpFPs, native N and C termini are fused by a short linker, and the new N and C termini are created near the chromophore. Exploiting the new N and C termini, the cpFPs is usually inserted between two units (two loops, or two domains) of a substrate-binding protein, in a way that structural changes induced by the analyte binding will reflect in a change of the fluorescence intensity [181].

The BiFC system is based on the possibility to split fluorescence proteins into two fragments, so that they can reform the complete β-barrel, recovering their fluorescence [182]. The components of the sensing domain are fused to the two halves of the fluorescence protein and are joined under the cognate stimulus, resulting in the appearance of fluorescence [183]. Finally, genetically encoded FRET sensors consist of a sensing domain flanked by two FPs, donor and acceptor, whose distance and/or relative orientation is affected by biological interaction leading to a FRET variation [179].

In the last years, in addition to a wide range of GFP-like proteins, NIR-FPs are emerging for biosensor design [184]. NIR-FPs, mainly engineered from bacterial phytochromes (BphPs), belong to the photoreceptor superfamily and have maximum absorbance at about 650–700 nm. They are not fluorescence by themselves, but their spectral properties depend on a covalently attached chromophore, biliverdin IXα (BV). BphPs have raised great attention, since BV is a catabolic metabolite of heme, naturally abundant in mammalian cells and tissues, thus allowing the photo-switching in vivo, without external addition of the chromophore. Furthermore, the modular domain structure of BphPs, is advantageous for the development of split- or insertion-based NIR biosensors [105,185,186].

Hybrid-systems, also known as chemical-genetic reporters, are based on the same principle as NIR-FPs, but they require an exogenous chromophore [187]. Schematically, they rely on a genetically encoded part that recognizes a specific fluorogen, that is dark when free, and bright only when bound. These reporters are favorable in terms of imaging contrast and, they do not subtract natural chromophores. Due to the progress of modern chemistry, the spectral and physicochemical properties of synthetic dyes are expanding, producing not only different colors (ranging from Vis to NIR) but also molecules permeable and not permeable to cell membrane. Chemical-genetic reporters include different systems, allowing covalent or non-covalent binding of the fluorescence molecules. Among covalent binding systems, the most popular ones are the self-labeling proteins SNAP-Tag, CLIP-tag, and Halo-Tag, and the recently reported photoactive yellow protein (PYP-tag). On the contrary, non-covalent binding systems include fluorogen-activating proteins (FAPs) and fluorescence-activating and absorption-shifting tag (FAST) technologies [187].

Finally, exogenous fluorescence agents, encompassing small molecule dyes and nanoparticles (organic and inorganic) developed in the visible as well as in the NIR-range, have also found application in molecular sensing. These agents can be distinguished in “always on” probes, largely used as tags (for labeling of antibodies, receptors, proteins, or specific molecular recognition elements), and applied mainly for bioimaging [188].

On the other hand, “turn-on” (also known as activatable or smart) probes switch their fluorescence on, or modulate dual emission ratio (radiometric probes), in response to specific stimuli such as changes in microenvironment indicators (pH, ions, hypoxia, reactive oxide, and reactive sulfur species, etc.) or biological targets (tumor receptor, antigens, etc.) (see Figure 4).

Hereafter in the next paragraphs we will report recent applications, using the above approaches, for sensing of calcium, heavy metal ions, nicotinamide adenine dinucleotide (NAD^+^), pH, neurotransmitters, and reactive oxide species (Table 2).

The calcium ion (Ca^2+^) is an important signaling molecule implicated in many cellular processes, and the remodeling of Ca^2+^ homeostasis is a feature of a variety of pathologies, including cardiovascular disease, neurological disorders, and cancer [189]. A widespread group of optical sensors for in vivo Ca^2+^ has been reported, based on cpGFP-like proteins (GCaMP, GECOs series), or on FRET (Camaleon series) [190]. Recently, the group of R.P. Campbell has built the first NIR-genetically encoded calcium indicator for optical imaging (NIR-GECO1), based on the insertion of the Ca^2+^-binding domain (Calmodulin (CaM)-RS20) into monomeric infrared fluorescence protein (mIFP), such that Ca^2+^ binding influence the BV chromophore environment and the fluorescence intensity. NIR-GECO1 undergoes a 90% decrease in fluorescence intensity upon Ca^2+^ binding, and its performance was evaluated in cultured neurons and in vivo, expressing the gene in the mouse motor cortex [191]. Another NIR calcium indicator with a positive response and high affinity has been obtained with a single domain near-infrared fluorescence protein (GAF-FP) and calmodulin/M13-peptide pair (GAF-CaMP2). The authors, by the fusion of the GAF-CaMP2 with super-folder GFP (sfGFP), produced also a ratiometric sensor, that was applied for the simultaneous visualization of calcium transients, in three organelles of mammalian cells using four-colors fluorescence microscopy [192,193].

In addition, chemical-genetic sensors for calcium have been developed. They are based on cpFAST [194] and split-FAST [195]. A cpFAST able to form green-yellow fluorescence complexes with 4-hydroxy-3-methylbenzylidene rhodamine (HMBR), or orange-red complexes with 4-hydroxy-3,5- dimethoxybenzylidene rhodanine (HBR-3,5-DOM), was connected to the M13 peptide (at the N-terminus) and to calmodulin (at C-terminus). The presence of Ca^2+^ promoted fluorogenic binding and the obtained sensor displayed dissociation constants for Ca^2+^ of about 60–100 nM [194]. The Split-FAST sensor is a reversible system, able to monitor rapid Ca^2+^ transient levels [195].

Finally, a novel turn-on fluorescence probe for calcium displayed a rapid enhancement of fluorescence emission (at 525 nm) in response to Ca^2+^ both in solution (detection limit 2.70 × 10^−7^ M) and in living cells (HeLa cells) [196].

Among metal ions, particular attention is pointed towards the detection of mercury, and its organic form methylmercury, due to their in vivo toxicity [197,198]. Different NIR cell-permeable tricarbocyanine dyes (IR-897, IR-877, and IR-925), have been used to detect Hg^2+^ and MeHg^+^ in living cancer cell lines [156]. In another example, a NIR three channels fluorescence probe (HCy-she) has been applied for simultaneously monitoring of the O_2_^−^ and Hg^2+^ in chronic mercury poisoning mouse models, and in HEK293 cells, since it is known that the toxic effect of Hg^2+^ and MeHg is mediated by O_2_ production [199].

An interesting in vivo FRET sensor for MeHg^+^ was based on heptamethine cyanine dye hCy7 (as acceptor) conjugated to Up Conversion Nanoparticles (UCNPs as donors). In this sensor, in the absence of MeHg^+^, UCNPs covered with hCy7 exhibits the quenching of their fluorescence emission at 660 nm; following the binding of MeHg^+^ hCy7 dye showed a red shifting in its absorption peak (from 670 to 845 nm) thus quenching the UCNP fluorescence emission at 800 nm. By measuring the ratio of the red and NIR fluorescence emission intensities, the authors obtained a radiometric sensor, that was able to detect the presence of MeHg^+^ in mouse tissues, when in vivo injected (LoD of 0.18 ppb in solution) [200].

In addition to these exogenous probes-based sensors, bacterial phytochromes (IFP1.4) have also been exploited for a genetically encoded mercury sensor. Binding of heavy metal ions to cysteine involved in the BV binding competes with formation of a covalent bond with BV, resulting in a loss of fluorescence. The sensitivity of IFP1.4 to mercury was higher in vitro (IC50% of 50 nM) compared to those found in mammalian cells (IC50% of 32 μM) [201].

Understanding of NAD^+^ metabolism provides many critical insights into health and diseases. Its levels are often altered in aging, neurodegeneration, kidney injury, obesity, diabetes, adipogenesis, cancer, and congenital malformations [202,203]. Several genetically encoded sensors able to detect the NADH/NAD^+^ ratio in living cells have been reported [204,205], but the absolute quantification of NAD^+^ has only recently been obtained by Cambronne et al. [206] who reported a radiometric sensor based on bacterial DNA-ligase and cpVenusFP (LigA-cpVenus), showing fluorescence reduction upon NAD^+^ binding. Very recently, Zou et al. [207] managed to obtain a sensor lighting in response to NAD^+^ (FiNAD). “FiNad” is based on the insertion of cpYFP into the NAD^+^/NADH binding domain of the bacterial transcription repressor protein (T-Rex), optimized in order to recognize specifically NAD^+^. This sensor was able to monitor NAD^+^ metabolism in a variety of organisms, including bacteria, cell lines, mice, zebrafish, and human-derived stem cells. In addition, Sallin et al. [208] introduced a new class of semisynthetic biosensors for the quantification of free NAD^+^, both in vitro (as a point of care assay) and in live cells. This bioluminescence resonance energy transfer (BRET) sensor exploits the capability of fluorescent derivatives of sulfamethoxazole to bind to human sepiapterin reductase (SPR) in a NAD^+^ dependent manner. The sensor consists of the SPR as analyte-binding protein fused to the self-labeling protein SNAP-tag and the luciferase NanoLuc (Nluc). When a synthetic tethered ligand is added, BRET from NLuc to Cy3 is possible, and the BRET efficiency increases linearly with the NAD^+^ levels [208].

Neurotransmitters, such as dopamine and acetylcholine (ACh) play a pivotal role in many physiological and pathological processes, and their reliable and specific spatiotemporal monitoring is a challenging goal, especially in animals executing complex behaviors. Recently, Jing M et al. [209] have, for the first time, developed a G Protein-Coupled Receptor (GPCR)-based sensor for ACh, with an approach that can be expanded to image other neurotransmitters and/or neuromodulators. In particular, they incorporated circularly permuted enhanced green fluorescent protein (cpEGFP) to the intracellular loop of human muscarinic ACh GPCRs. They used these sensors to probe ACh dynamics both in vitro and in vivo in mice and transgenic flies. Similarly, Sun et al. [210] have reported two DA sensors engineered by coupling a cpEGFP to a selected human GPCR dopamine receptor. These sensors, with different DA affinities, allowed for a real-time detection of endogenous extracellular DA in acute brain slices of mice, and in the intact brains of versatile animal models including flies, fish, and mice. Building upon the same strategy, Feng and colleagues [211] inserted cpEGFP into the beta-2 adrenergic GPCR for sensing epinephrine (NE) in vivo.

The reactive oxygen radicals as hydrogen peroxide (H_2_O_2_), superoxide anion radical (O_2_^−^), hydroxyl radical (HO^−^), peroxynitrite (ONOO^−^), and nitric oxide (NO) play important roles in both physiological and pathological conditions, increasing in some diseases, such as cancer, and inflammation. The in vivo detection is difficult due to a short half-life, but different kinds of sensors have been also reported [212]. Genetically encoded sensors for ROS, in particular for H_2_O_2_ (namely HyPer) consist of a circularly permuted yellow fluorescence protein (cpYFP) inserted into the regulatory domain of the *Escherichia coli* hydrogen peroxide-binding protein (OxyR). Hyper is an H_2_O_2_^−^ specific ratiometric, and therefore quantitative, sensor that has been largely applied for in vivo studies [213]. Peroxynitrite (ONOO^−^)-activatable NIR II FRET probes have been recently reported, exhibiting the wavelength tunability of cyanine dyes. These probes have been applied for bioimaging of ONOO, measured as biomarker of drug (acetaminophen) induced hepatotoxicity in vivo [214]. An additional specific redox species involved in the drug hepatotoxicity, is represented by Nitric Oxide (NO), detected by an activatable organic semiconducting nanoprobe (AOSNP). The presence into the cells of NO induces a shift of the fluorescence from the NIR-I region to the NIR-II region, in a NO-sensitive organic semiconducting group (FTBD) injected in live mice [215]. An alternative Vis-FRET assay was used for nanomolar detection of NO. In absence of NO, the probe consisting in a donor, FITC, and an acceptor, DABCYL, linked via 1,4-dihydropyridine, does not emit light, because the acceptor quenches the fluorescence emitted by FITC. In presence of the NO, the linker is broken and the fluorescence can be detected [216].

Physiological and pathological cell processes are accompanied by pH changes, at both extracellular, cellular, and subcellular compartments [217,218]. In vivo pH sensing has been achieved by different systems, ranging from genetically encoded fluorescence proteins sensitive to pH changes by themselves [219] to turn-on probes [220], and also semi-synthetic systems [221]. Tang et al. [222] described a probe sensitive to the difference in hydrogen ion concentrations in living cells, by synthesizing a 3-aminophenol into the parent nucleus of indole heptamethine cyanine dye. The authors, by phagocytosis experiments, demonstrated that the probe easily penetrates the cells and fluorescence intensity increased within the pH range values of 4.0–6.5. Different NIR fluorescence probes have been studied for tumor analysis since in tumor cells, the pH is very acidic [223,224,225]. An example is indocyanine green derivatives, changing their loop structure from open to closed, in response to pH ranges from, 7–9, 5–7, and 3–6 [226]. The pH is also a fundamental factor for the digestive process, and it must be kept constant in the stomach. A low-pH sensor for quantitative measurement of gastric pH in vivo was realized, based on an anti-quenching pentamethine cyanine fluorophore, called BTC1070, exhibiting fluorescence in response to low pH values [227].

A new semisynthetic sensor has been recently reported by Perkins et al. [221] and relies on Fluorogen-Activating Peptide (FAP) technology coupled to pH-sensitive probes. This sensor consists of tandem dye molecules formed by a Cy3 (donor) linked to a fluorogenic malachite green (acceptor), that are targeted and activated upon binding to a FAP, on the cell surface. Since Cy3 is pH sensitive, when exited, it emits only in a limited range of pH, thus allowing pH-specific FRET activation. Upon internalization, the FRET-based emission ratio of the biosensor can distinguish pH values in different cell compartments, and it is suitable for the analysis of protein trafficking through pH values changes associated with endo- and exocytosis.

## 4. Conclusions

SPR- and fluorescence-based biosensors represent powerful tools for the detection of a large number of analytes that fall into fields of high social and economic interests, such as food safety, environmental control, national security, and health.

In this review we have reported several illustrative examples of recent applications of optical biosensors, discussing what could be the impact of biosensors not only in biomedical research but also in several aspects of our daily life. SPR- and fluorescence-based biosensors have rapidly evolved, and their technological progresses are still in expansion, improving their performance in terms of precision, multiplexing, and possibilities for in vivo imaging.

SPR methodologies (SPR-ATR, EC-SPR, LSPR, SPRi, PSPR, and SPRM) were generated from different configurations in terms of geometry arrangements, light source, detection methods, and sensor surface and all of them have the indisputable advantage of being label-free and real-time methodologies. They are characterized by high sensitivity, rapidity, and cost-effective while retaining the conformational and functional integrality of biomolecules to be investigated. In addition, recent advances in multiplex applications, high throughput arrays, miniaturization, and signal enhancements using noble metal nanoparticles, promise the achievement of unprecedented sensitivity (up to the level of single-molecule detection) and the application of SPR-based sensors in point-of-care testing platforms. Nevertheless, SPR has intrinsic limitations due to sensing on chip surfaces, with challenges connected to immobilization strategies and sensing element-target binding kinetics. In addition, despite progresses that are in course, SPR technology is hardly able to detect small molecular weight analytes.

On the contrary, fluorescence-based biosensors, are highly sensitive and with a wide dynamic range, while allowing rapid analysis, in solution. Among different technological improvements, they have taken great advantages from the development of nanoparticles, that have become important fluorescence probes for both in vitro and in vivo bioimaging. Indeed, examples of nanoparticle-based sensors are reported throughout all the fields of applications that we have presented. Furthermore, the growing availability of NIR fluorescence labels has supported the production not only of sensors for direct analysis in real matrices, in vitro, but also of new in vivo sensors, outperforming previous reporters in terms of spatial and temporal resolutions, at single-cell, tissue, and even full-body levels. Although the research proceeds rapidly, several issues remain to be optimized. A general disadvantage of the use of fluorescence can derive from the process of labeling of probes and/or targets, that requires additional working procedures for the sensor building. In addition, this process could also affect the native properties of the molecules, thus affecting probe-target interactions. Furthermore, especially for in vivo biosensors, specific limits should be considered, such as the signal to noise ratio, or troubles in the expression efficiency of genetically encoded sensors.

Research is still very active to overcome general and specific limits, as well as to create complete biosensors that can be introduced on the market. In this regard, the production of a mature biosensor device requires the cooperation between different disciplines, to achieve the integration of the biosensing element into a platform (such as portable SPR, portable fluorometers, smartphones) that fulfills methodological and practical aspects such as robustness, reproducibility, simplicity and shelf life. For this reason, the large practical application of biosensors discussed in this review requires additional research efforts, and it is still in its beginning. Further developments of different research areas, coupled with the versatility of SPR and fluorescence-based methodologies, will positively impact the production of complete biosensors, promising in the next future, to improve important aspects of the human quality of life.

## Figures and Tables

**Table 1:** List of analyte targets detected by surface plasmon resonance (SPR)-based sensors.

Analyte	Method	Substrate/Sensing Layer	LoD	Ref.
Food				
*Campylobacter jejuni*	SPR-ATR	Au-coated thin glass/Antibody	1.0 × 10^3^ CFU/mL	[33]
*“ ”*	SPR-ATR	Au-coated thin glass/Antibody	1.1 × 10^5^ CFU/mL	[38]
*Salmonella typhimurium*	SPR-ATR	C18 Au-coated thin glass/Antibody	2.5 × 10^5^ CFU/mL	[34]
*“ ”*	SPR-ATR	Au-coated thin glass/Antibody	1.0 × 10^5^ CFU/mL	[35]
*Salmonella enteritidis*	SPR-ATR	C18 Au-coated thin glass/Antibody	2.5 × 10^8^ CFU/mL	[34]
*“ ”*	SPR-ATR	Au-coated thin glass/Antibody	1.0 × 10^6^ CFU/mL	[37]
*Salmonella choleraesuis*	SPR-ATR	Au-coated thin glass/Antibody	4.4 × 10^4^ CFU/mL	[38]
*Escherichia coli* O157:H7	SPR-ATR	Au-coated thin glass/Antibody	1.0 × 10^5^ CFU/mL	[35]
*“ ”*	SPR-ATR	Au-coated thin glass/Antibody	1.4 × 10^4^ CFU/mL	[38]
*Yersinia enterocolitica*	SPR-ATR	Au-coated thin glass/Antibody	1.0 × 10^5^ CFU/mL	[35]
*Legionella pneumophila*	SPR-ATR	Au-coated thin glass/Antibody	1.0 × 10^5^ CFU/mL	[35]
*Listeria monocytogenes*	SPR-ATR	Au-coated thin glass/Antibody	1.0 × 10^7^ CFU/mL	[36]
*“ ”*	SPR-ATR	Au-coated thin glass/Antibody	1.0 × 10^6^ CFU/mL	[37]
*“ ”*	SPR-ATR	Au-coated thin glass/Antibody	3.5 × 10^3^ CFU/mL	[38]
Aflatoxin B_1_	SPR-ATR	Au-coated thin glass/Antigen	0.2 ng/gr	[39]
Fumonisin B_1_	SPR-ATR	Au-coated thin glass/Antibody	50 ng/mL	[40]
Enterotoxin B	SPR-ATR	Au-coated thin glass/Antibody	100 fM	[41]
Ricin	SPR-ATR	Au-coated thin glass/Antibody	200 ng/mL	[42]
Abrin	SPR-ATR	Au-coated thin glass/Antibody	75 ng/mL	[43]
Tetrodotoxin	SPR-ATR	Au-coated thin glass/Antigen	0.3 ng/mL	[44]
Tylosin	SPR-ATR	Au-coated thin glass/Antigen	2.5 µg/Kg	[45]
Phenol	SPR-ATR	Au-coated thin glass/Antigen	5 ppm	[46]
Ascorbic acid	SPR-ATR	Fiber optic core/Ag PANI MIP	1.28 × 10^−^^10^ M	[47]
Environment				
Chlorpyrifos	EC-SPR	Au-coated thin glass/MIP Fe_3_O_4_-PDA NPs	0.76 nM	[48]
Atrazine	SPR-ATR	Fiber optic core/Ag MIP	1.92 × 10^−14^	[50]
VOCs (1-octanol)	SPRi	Au-coated thin glass/Biomimetic peptides	375 ppb	[51]
Pb^2+^	SPR-ATR	Au-coated thin glass/Ag-CS	30 ppb	[52]
*“ ”*	SPR-ATR	Au-coated thin glass/Ag-CS-GO	30 ppb	[53]
Co^2+^	SPR-ATR	Au-coated thin glass/PAR-Cs-GO	10 ppb	[54]
Cu^2+^	SPR-ATR	Au-coated thin glass/CTA-NCC-GO	0.01 ppm	[55]
Security				
TNT	SPR-ATR	Au-coated thin glass/Antigen	0.002 ng/mL	[57]
*“ ”*	SPR-ATR	Au-coated thin glass/PAMAM-antigen	110 pg/mL	[58]
*“ ”*	SPR-ATR	Fiber optic core/Au MIP	5.1 × 10^−5^ M	[59]
Capsaicinoids	SPR-ATR	Au-coated thin glass/OEG-antigen	150 ppb	[60]
Homovanillic acid	SPR-ATR	Au-coated thin glass/Apten antigen	150 ppb	[61]
Health				
Newcastle disease virus	LSPR	Fiber optic Ex-TFGs /Au-NP-Antibody	25 pg/mL	[62]
HIV-1 virus	LSPR	Au NP-coated thin glass/Antibody	200 fg/mL	[63]
Dengue virus	SPR-ATR	Au-coated thin glass/Antibody	2 × 10^4^ particles/mL	[64]
Avian influenza A H7N9 virus	SPR-ATR	Au-coated thin glass/Antibody	402 copies/mL	[65]
Progesterone	SPR-ATR	Au-coated thin glass/Antigen	170 pg/mL	[66]
Estradiol	SPR-ATR	Au-coated thin glass/Antigen	3.5 ng/mL	[67]
Cortisol	SPR-ATR	Au-coated thin glass/OEG-antigen	49 pg/mL	[68]
Testosterone	SPR-ATR	Au-coated thin glass/OEG-antigen	15.4 pg/mL	[69]
Sulfamethazine	SPR-ATR	Au-coated thin glass/Antigen	0.015 µg/mL^−1^	[70]
Sulfadiazine	SPR-ATR	Au-coated thin glass/Antigen	0.052 µg/mL^−1^	[70]
Clenbuterol	SPR-ATR	Au-coated thin glass/Antigen	0.4 ng/mL^−1^	[71]
Ethinylestradiol	SPR-ATR	Au-coated thin glass/Antigen	0.5 ng/mL^−1^	[71]
Enrofloxacin	SPR-ATR	Au-coated thin glass/Antigen	1.2 ng/mL^−1^	[71]
Tetracycline	SPR-ATR	Fiber optic core/Ag MIP	0.01 µM	[72]
*“ ”*	SPR-ATR/LSPR	Fiber optic core/Ag NP/MIP	2.2 × 10^−9^ M	[73]
Oxytetracycline	SPR-ATR	Fiber optic core/Ag MIP	0.01 µM	[72]
Erythromycin	SPR-ATR	Fiber optic core/Ag MIP	6.2 × 10^−8^ M	[74]
Vitamin B_3_	SPR-ATR	Fiber optic core/Ag MIP	0.5 mg/mL	[75]
Nicotine	SPR-ATR	Fiber optic core PMMA/Au MIP	1.86 pM	[78]
Epstain-Barr Virus	SPR-ATR	Au-coated thin glass/Antigen	0.1 ng/mL	[81]
Interleukin-6	SPR-ATR	Fiber optic core/Antibody	0.92 ng/mL	[82]
Prostate-specific antigen	SPR-ATR	Au-coated thin glass/Antibody	8.5 pM	[84]
Carbohydrate antigen 15-3	SPR-ATR	Au-coated thin glass/Antibody	0.025 U/mL	[85]
Carcinoembryonic antigen	EC-SPR	Au-coated thin glass/Antibody	0.5 ng/mL^−1^	[86]
C-reactive protein	SPR-ATR	Au-coated thin glass/Antigen	0.1 ng/µl	[87]
HER2	SPR-ATR	Au-coated thin glass/Antibody	11 ng/mL	[88]
Progesterone receptor	SPR-ATR	Au-coated thin glass/Antigen	3.56 ng/mL	[90]
Metalloproteinases-9	SPR-ATR	Au-coated thin glass/Antibody	8 pg/mL	[91]
BRCA1	SPRi	Au-coated thin glass/Au NPs/DNA	1 pM	[92]
Cholesterol	SPR-FTIR	Au-coated thin glass/Cells	9 mg/gr	[99]
Volatile compound (octanal)	SPRi	Au-coated thin glass/Cells	0.1 mM	[100]

**Table 2:** List of analyte targets detected by fluorescence-based sensors.

Analyte	Method	Sensing Element/Fluorescent Molecules	LoD/Dynamic Range	Ref.
Food				
**Penicillin G**	FP	Antibody/NIR-CF647	1.0 nmol/L	[134]
**Ciprofloxacin**	FP	Antibody/NIR-CF647	1 ppb	[135]
**Amikacin**	FS	Enzyme/fluorescein-5 maleimide	40 nM	[122]
**Kanamycin A**	FS	Enzyme/fluorescein-5 maleimide	50 nM	[122]
**Gentamicin**	FS	Enzyme/fluorescein-5 maleimide	10 nM	[122]
**Neomycin**	FS	Enzyme/fluorescein-5 maleimide	7 nM	[122]
**Tobramycin**	FS	Enzyme/fluorescein-5 maleimide	5 nM	[122]
**Paromomycin**	FS	Enzyme/fluorescein-5 maleimide	10 nM	[122]
**Fluoroquinolone**	FP	Antibody/nanoparticles	0.1 nM	[123]
**Sulfadimethoxine**	FI	Aptamer/fluorescein amidite	10 ng/mL	[124]
**Chloramphenicol**	TR-FIA	Antibody/europium	0.05 ng g^−1^	[130]
**Fluoroquinolone**	TR-FIA	Antibody/Europium	0.053 μg/L	[131]
**Patulin**	FP	Antibody/NIR DyLight IF800	0.06 μg/L	[136]
**Ochratoxin A**	FCS	Antibody/FITC	0.0078 ng	[137]
**17β-estradiol**	FP	Antibody/CF647	<10 pmol	[140]
**Hexestrol**	FP	Receptor/coumestrol	2.94 nM	[141]
**Dienestrol**	FP	Receptor/coumestrol	2.89 nM	[141]
**Diethylstilbestrol**	FP	Receptor/coumestrol	3.12 nM	[141]
**α-lactalbumins**	FLISA	Antibody/CdSe/ZnS quantum dots	0.1 ng/mL	[142]
**Lactoferrin**	FP	Aptamer/FITC and Ag10NPs	1.25 pM	[143]
**Gluten**	FCS	Antibody/FITC	0.006 ppm	[144]
Environment				
**Testosterone**	TIRF	Antibody	0.2 ng/L	[147]
**Carbaryl**	FI	Enzyme/silicon quantum dots (SiQDs)	7.25 × 10^−9^ g/L	[155]
**Parathion**	FI	Enzyme/silicon quantum dots (SiQDs)	3.25 × 10^−8^ g/L	[155]
**diazinon**	FI	Enzyme/silicon quantum dots (SiQDs)	6.76 × 10^−8^ g/L	[155]
**Phorate**	FI	Enzyme/silicon quantum dots (SiQDs)	1.90 × 10^−7^ g/L	[155]
**Acetylcholinesterase**	FI	Enzyme/nanoclusters DNA-Cu/AgNCs	0.05 U/mL	[157]
**Acetylcholinesterase**	FI	Enzyme/nanoclusters AuNCs	2.0 × 10^−6^ U/mL	[158]
**DDVP**	FI	Enzyme/nanoclusters AuNCs	13.67 pM	[158]
**Benzene**	FRET	Protein/ 1-aminoanthracene	3.9 μg/m^3^	[160]
Security				
**Ricin A**	FI	Protein/AuNCs	400 nM	[164]
**Bacillus anthracis**	FI	Antibody/silicon nitride surfaces	10^3^ CFU/mL	[165]
Health				
**IL-1β**	FI	Antibody/FITC	8.91 pg/mL	[172]
**IL-6**	FI	Antibody/FITC	1.33 pg/mL	[172]
**IL-10**	FI	Antibody/FITC	6.12 pg/mL	[172]
**Dopamine**	FI	Bi-functionalized carbon dots	0.1 pM	[176]
**17β-estradiol**	FI	Aptamer/fluorescent dye	2.1 nM	[177]
**17β-estradiol**	FI	Antibody/nanoparticles DC-AuNPs	6.4 × 10^−6^ ng/mL	[178]
**Calcium ion**	FI	Turn-on fluorescent probe	2.70 × 10^−7^ M	[196]
**Calcium ion**	FI	Receptor/mIFP	0.01 to 1 μM ^a^	[191]
**Calcium ion**	FI	Receptor/GAF-sfGFP	0.1 to 5 μM ^a^	[193]
**Calcium ion**	FI	Receptor/cpFAST	0.04 to 0.2 μM ^a^	[194]
**Hg^2+^**	FI	NIR-activatable probe-HCy−SeH	10 to 60 μM ^a^	[199]
**Hg^2+^**	FI	BPs (IFP1.4)	32 μM	[201]
**MeHg^+^**	LRET	UCNPs/UCL	0.18 ppb	[200]
**O_2_-**	FI	NIR-activatable probe-HCy−SeH	10 to 60 μM ^a^	[199]
**NAD^+^**	FI	Enzyme/cpVenus	30 μM to 1 mM ^a^	[206]
**NAD^+^**	FI	Enzyme/cpYFP	0.1 to 10 mM ^a^	[207]
**NAD^+^**	BRET	Enzyme-cpLuc/Cy3	10^−4^ to 10^−8^ M ^a^	[208]
**Acetylcholine**	FI	Receptor/cpEGFP	10 nM to 100 μM ^a^	[209]
**Dopamine**	FI	Receptor/cpEGFP	10^−6^ to 10^−8^ M ^a^	[210]
**Epinephrine**	FI	Receptor/cpEGFP	10^−6^ to 10^−8^ M ^a^	[211]
**H_2_O_2_**	FI	Enzyme/NeonOxIrr	pH 5.5 to 7.5 ^a^	[192]
**Nitric Oxide**	FRET	FITC and DABCYL	100 pM to 5 nM ^a^	[216]
**hydrogen ion**	FI	Pentamethine cyanine fluorophores	pH 1.0 to 4.0 ^a^	[227]

^a^ The working dynamic range values are reported for in vivo biosensors.

## Data Availability

Data sharing is not applicable to this article.

## Abbreviations

The following abbreviations are used in this paper:

1-AMA	1-AMinoAnthracene
ACh	Acethylcoline
AChE	AcetylCholinEsterase
AFM1	Aflatoxin M1
AL	Amide-Linked
AOSNP	Activatable Organic Semiconducting NanoProbe
ATC	AcetylThioCholine
ATR	Attenuated Total Reflection
Au/CS/GO	gold-Chitosan-Graphene Oxide
BiFC	Bimolecular Fluorescent Complementation
BphPs	Bacterial Phytochromes
BRET	Bioluminescence Resonance Energy Transfer
BSA	Bovin Albumin Serum
BTEX	Benzene, Toluene, Ethyl-benzene and Xylene
BV	BiliVerdin
CA15-3	Carbohydrate Antigen 15-3
CCD	Charge-Coupled Device
CCH	oncholepas oncholepas Hemocyanin
CEA	CarcinoEmbryonic Antigen
CFU	Colony-Forming Unit
CMA	Cow Milk Allergy
CpEGFP	circularly permuted Enhanced Green Fluorescent Protein
CPF	ChlorPyriFos
CpFPs	circularly permuted Fluorescent Proteins
CpYFP	circularly permuted Yellow Fluorescent Protein
CRP	C-Reactive Protein
CS–GO	Chitosan–Graphene Oxide
CTA-NCC	hexadeCyltrimeThylAmmonium bromide-NanoCrystalline Cellulose
CWAs	Chemical Warfare Agents
DA	DopAmine
DDVP	Dimethyl-Di-chloroVinyl Phosphate
DNA	DeoxyriboNucleic Acid
DNA-Cu/AgNCs	DNA-templated copper/silver nanoclusters
EBV	Epstain Bain Virus
EC-SPR	Electrochemical Surface Plasmon Resonance
eGFP	enhanced Green Fluorescent Protein
ELISA	Enzyme-Linked ImmunoSorbent Assay
ER	Estrogen Receptor
ER-LBD	Estrogen Receptor α-Ligand Binding Domain
ERY	Erythromycin
Ex-TFG	Excessively tilted fiber grating
FAP	Fluorogen Activating Peptide
FAST	Fluorescence-Activating and Absorption-Shifting Tag
FCS	Fluorescence Correlation Spectroscopy
FEP	Fluorinated Ethylene Propylene
FI	Fluorescence Intensity
FITC	Fluorescein IsoThioCyanate
FLISA	Fluorescence-Linked Immunosorbent Assay
FP	Fluorescence Polarization
FRET	Förster Resonance Energy Transfer
FT-IR	Fourier-transform infrared spectroscopy
FTIR-SPR	Fourier Transform Infra-Red Surface Plasmon Resonance
GO	Graphene Oxide
GPCR	G Protein Coupled Receptor
HER2	Human Epidermal growth factor Receptor 2
HMBR	4-hydroxy-3-methylbenzylidene rhodanine
IM-SPR	Intensity-Modulated Surface Plasmon Resonance
InA	Internalin A
Lac	Lactoferrin
LoD	Limit of Detection
LSPR	Localized Surface Plasmon Resonance
MBL	Metal-Binding Loop
mIFP	monomeric Infrared Fluorescent Protein
MIP	Molecular Imprinting Polymer
MIPs	Molecular Imprinting Polymers
MIT	Molecular Imprinting Technology
MMP-9	Matrix Metalloproteinases-9
MMPs	Magnetic MicroParticles
MRE	Molecular Recognition Element
MRL	Maximum Residual Limit
NAD	Nicotinamide Adenine Dinucleotide
NC	Nanocluster
NDV	Newcastle Disease Virus
NHS	N-hydroxy Succinimide
NIR	Near InfraRed
NIR-FP	Near InfraRed Fluorescent Protein
NO	Nitric Oxide
NPs	nanoparticles
OBP	odorant binding protein
OEG	Oligo-Ethylene Glycol
OTC	Oxytetracycline
PAMAM	PolyAMidoAMine
PANI	polyaniline
PAT	Patulin
PBS	Phosphate Buffered Saline
PCs	Photonic Crystals
PenG	Penicillin G
PR	Progesterone Receptor
PSA	Prostate-Specific Antigen
PSPR	Propagating Surface Plasmon Resonance
PYP	Photoactive Yellow Protein
QD	Quantum Dot
RI	Refractive Index
SAMs	Self Assembled Monolayers
SECM	Scanning EleCtrochemical Microscopy
SELEX	Systematic Evolution of Ligands by EXponential enrichment
sfGFP	superfolder Green Fluorescent Protein
SiQD	Silicon Quantum Dots
SNP	Single-Nucleotide Polymorphism
SPME	Solid-Phase Microextraction
SPR	Surface Plasmon Resonance
SPRi	Surface Plasmon Resonance imaging
SPRM	Surface Plasmon Resonance Microscopy
SWNT	Single-Walled carbon NanoTube
TC	TetraCycline
TIR	Total Internal Reflection
TIRF	Total Internal Reflectance Fluorescence
TNT	Trinitrotoluene
tPSA	total Prostate-Specific Antigen
TR-FIA	Time-Resolved Fluorescence-ImmunoAssay
TTX	Tetrodotoxin
UCNPs	Up Conversion Nanoparticles
VOC	Volatile Organic Compound
VOCs	Volatile Organic Compounds
